# History of Oral Mucosal Lesions in Oral Squamous Cell Carcinoma Patients

**DOI:** 10.3290/j.ohpd.c_2028

**Published:** 2025-06-03

**Authors:** Arvi Keinänen, Johanna Snäll, Jaana Hagström, Johanna Uittamo

**Affiliations:** a Arvi Keinänen PhD Student, Department of Oral and Maxillofacial Diseases, University of Helsinki and Helsinki University Hospital, Helsinki, Finland. Made substantial contributions to conception and/or design of the work and contributed to the acquisition, analysis, and interpretation of data.; b Johanna Snäll Associate Professor, Department of Oral and Maxillofacial Diseases, University of Helsinki and Helsinki University Hospital, Helsinki, Finland. Made substantial contributions to conception and/or design of the work and contributed to the acquisition, analysis, and interpretation of data.; c Jaana Hagström Professor, Department of Pathology, University of Helsinki and Helsinki University Hospital, Helsinki, Finland, Research Programs Unit, Translational Cancer Biology, University of Helsinki, Helsinki, Finland; Department of Oral Pathology and Radiology, University of Turku, Turku, Finland. Made substantial contributions to conception and/or design of the work and contributed to the acquisition, analysis, and interpretation of data.; d Johanna Uittamo Adjunct Professor, Department of Oral and Maxillofacial Diseases, University of Helsinki and Helsinki University Hospital, Helsinki, Finland. Made substantial contributions to conception and/or design of the work and contributed to the acquisition, analysis, and interpretation of data.

**Keywords:** dysplasia, oral cancer, oral mucosal finding, oral squamous cell carcinoma.

## Abstract

**Purpose:**

To evaluate the occurrence of previous mucosal dysplasia in patients with oral squamous cell carcinoma (OSCC) and to charaterise patient profile, types of previous oral mucosal lesions, and care-seeking in relation to earlier mucosal findings.

**Materials and Methods:**

Retrospective data of OSCC patients with a primary tumour were collected. The primary outcome variable was any history of oral mucosal findings; the secondary outcome variable was a history of previous oral mucosal dysplasia. The primary predictor variable was the mode of seeking treatment. Patient and tumour-related variables were compared between patients with and without anamnestic mucosal changes or findings.

**Results:**

A total of 528 patients were included in the study. Of these patients, 169 (32.0%) had a history of an oral mucosal lesion. Oral mucosal dysplasia was detected in 34 patients (6.4%) before the OSCC diagnosis. Patients who had a history of heavy alcohol use were less likely to have a history of any mucosal lesions or dysplasia (adjusted odds ratio [aOR] 0.350, 95% confidence interval [CI] 0.215-0.571, p < 0.001 and aOR 0.235, 95% CI 0.070-0.795, p = 0.020). Tumours were detected more often in conjunction with routine appointments in patients with a history of any mucosal lesions (aOR 2.671, 95% CI 1.704-4.187, p < 0.001) and in those with previously detected dysplasia (aOR 6.195, 95% CI 3.004-12.774, p < 0.001).

**Conclusions:**

The results emphasise the importance of careful examination and close follow-up of findings in the oral mucosa.

Oral epithelial dysplasia is described as a growth anomaly produced by abnormal or atypical epithelial proliferation, resulting in a lesion with disturbed differentiation and maturation of epithelial tissue. These epithelial changes in the mucosa have a higher rate of developing into oral squamous cell carcinoma (OSCC) than does healthy mucosa.^11,23,39,41^ Clinically, dysplasia typically occurs as leukoplakia, erythroplakia, or erythroleukoplakia, but epithelial dysplasia is not always present in these benign lesions.^6,19,23^ Oral epithelial dysplasia is present in first biopsy in 40% of leukoplakia, 91% of erythroplakia, and  < 10% of proliferative leukoplakia lesions.^33,38^ Oral lichen planus has been considered a premalignant lesion, although controversy about its classification exists in the literature.^36^ Other known oral mucosa-related risk factors for OSCC are human papilloma virus, candida, trauma caused by ill-fitting prostheses or other continuous mechanical irritation, and possibly autoimmune polyendocrinopathy-candidiasis-ectodermal dystrophy syndrome.^1,5,8,15,30^

OSCC comprises 90% of all oral carcinomas.^13,37^ Smoking and alcohol consumption are the main risk factors for development of OSCC-preceding dysplasia.^23^ Of oral epithelial dysplasia, 12.1% develops into malignancy.^21,23^ The OSCC disease-specific survival beyond 3 years is approximately 70% in patients with stage I tumours, but only 60% in patients with stage III or IV tumours.^7^ The 5-year mortality rate of OSCC is close to 50%.^16^

Thus, for early OSCC diagnosis, the detection, diagnosis, and follow-up of mucosal changes are essential. However, different mucosal changes are found in the oral mucosa, some of which are clinically similar and may mislead clinicians.^42^ Thus, oral disease diagnostics is challenging, especially for conditions that require specialised medical care. According to an Italian study, 55% of the referrals for specialised medical care lacked a clinical diagnosis.^31^

This study focused on early oral mucosal dysplasia in patients with OSCC. We examined patient profiles, the types of early oral lesions, and how patients sought care based on these earlier findings. We hypothesised that patients with dysplasia are often diagnosed with OSCC during routine healthcare visits and at an earlier stage of the disease.

## MATERIALS AND METHODS 

This study was approved by the Internal Review Board of the Head and Neck Centre, Helsinki University Central Hospital, Finland (HUS/66/2018).

### Patient Material

Patient records from January 2016 to December 2020 at Helsinki University Hospital, Helsinki, Finland, were evaluated retrospectively. Patient data were retrieved from the multidisciplinary Head and Neck Tumour Board of Helsinki University Hospital, which maintains patient information on all patients treated in the university hospital region who have a primary diagnosis of OSCC.

### Inclusion and Exclusion Criteria

All patients with a primary OSCC diagnosis evaluated at Helsinki University Hospital were included in the study. Patients with a history of previous oral cancer were excluded.

### Study Design

The primary outcome variable was a history of earlier oral mucosal changes or findings. History of oral mucosal lesions was evaluated from patient records in the hospital database, which were based on anamnestic information and referral details. Oral mucosal lesions were divided into the following eight groups: lichenoid-type reaction (including lichen planus and lichenoid), leukoplakia, erythroplakia, clinically thickened epithelium, unspecific ulceration, inflammatory changes, papilloma, and unspecified mucosal findings (including benign findings without a specific description).^40^ Our secondary outcome variable was history of oral mucosal dysplasia.

The primary predictor variable was the mode of seeking OSCC treatment, defined as incidental (i.e., tumour noted in conjunction with routine care or control appointments) or other.

Explanatory variables were age, sex, smoking, heavy alcohol use, and tumour-related variables (tumour size, tumour site, and referring physician).

Patients were stratified by smoking status into non-smokers (non-smokers and former smokers who not smoked for ≥ 5 years) and current and former smokers (current smokers and former smokers who had not smoked for < 5 years).^17^ Alcohol use was determined according to the following Finnish Current Care Guidelines consumption limits for heavy alcohol use: ≥ 23 doses (≥ 287.5 g alcohol) per week for men and ≥ 12 doses (≥ 150 g alcohol) per week for women, as suggested by the Finnish Working Group for treatment of alcohol abuse.^14^ Tumour size was defined according to T categorisation as Tis-T2 (Tis, T1, or T2) or T3-4 (T3 or T4) based on TNM Staging of Lip and Oral Cavity cancers – AJCC 7th edition^10^ and 8th edition^2,22^ which were valid at the time of diagnosis.

### Statistical Analysis

Associations between explanatory and predictor variables and outcome variables were assessed with logistic regression analysis. The Hosmer and Lemeshow goodness-of-fit test was used to assess logistic regression analyses. Before conducting multiple logistic regression analyses, Cramer’s V-test was used to detect possible multicollinearity of categorical explanatory variables. The significance level was set at 0.05. SPSS 28.0 (IBM; Armonk, NY, USA) and used for all statistical analyses.

## RESULTS

### Patient Material

Of 682 evaluated OSCC patients, 154 were excluded (119 patients had previous oral cancer and 35 had missing data from preceding visits). Thus, 528 patients with primary OSCC were included in the final analyses.

**Fig 1:**
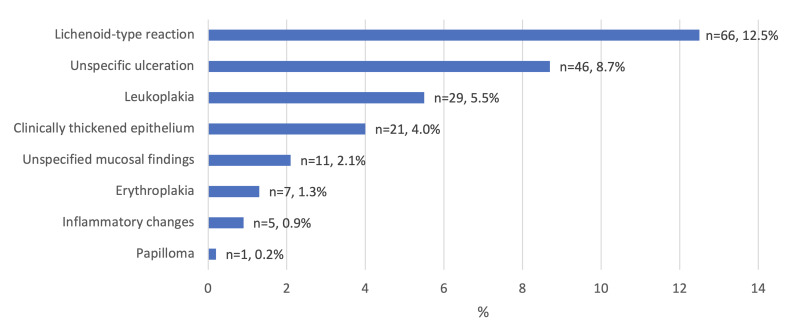
Previous oral mucosal findings of 528 oral squamous cell carcinoma patients. Of 169 patients with anamnestic oral mucosal changes or findings, 66 had a lichenoid-type reaction, including patients with a history of oral lichen planus (n = 48, 9.1%).

**Fig 2:**
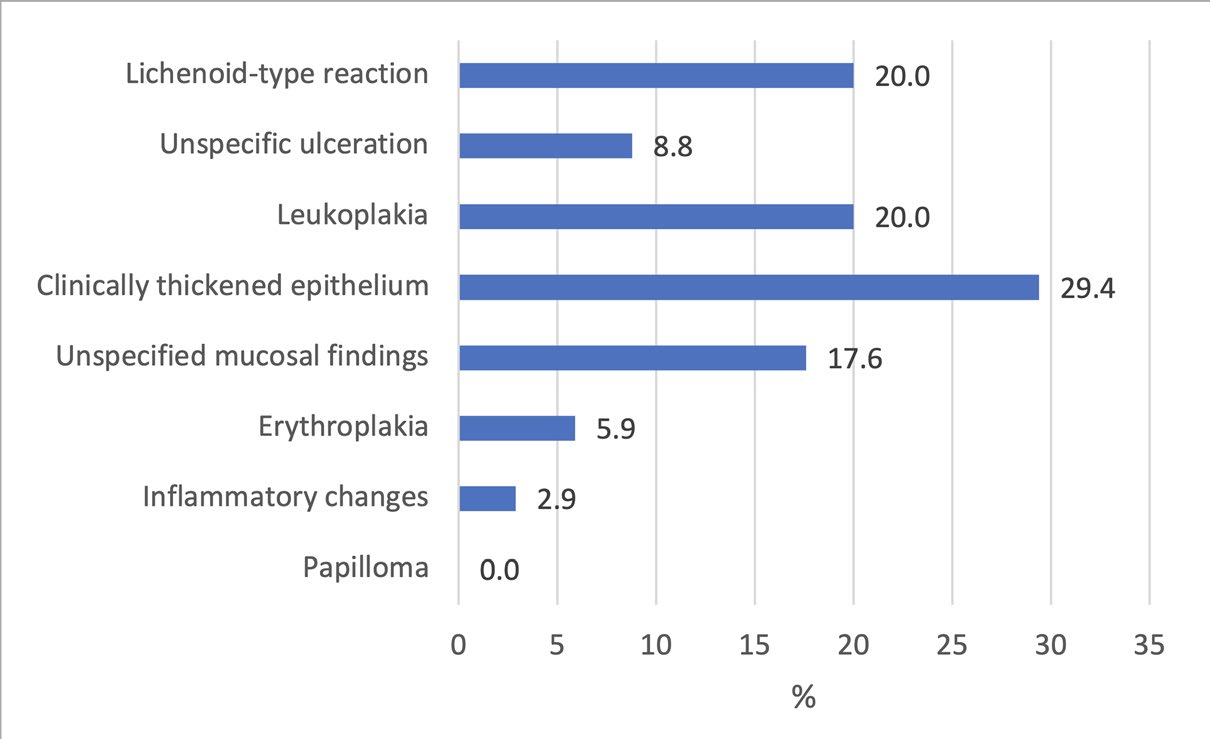
Previous oral mucosal findings of oral squamous cell carcinoma patients with dysplasia (n = 34).

Median patient age was 66.2 years. Patients were more often men (58.5%). Half of the patients were smokers (50.6%) and 26.7% reported a history of heavy alcohol use. In all, 169 of 528 patients (32.0%) had a history of oral mucosal lesion (Table 1). The types and occurrence of one or more earlier mucosal lesions are presented in Fig 1. Dysplasia was found prior to malignancy in 6.4% of OSCC patients. History of oral mucosal lesions in OSCC patients with previously found dysplasia are presented in Fig 2.

Univariate logistic regression analysis showed that OSCC in patients with an earlier oral mucosal finding was 2.6 times more likely to be found incidentally (p < 0.001). Moreover, a previously detected oral mucosal lesion was associated with female sex (odds ratio [OR] 2.183, p < 0.001). Earlier oral mucosal lesions were found statistically significantly less often in smokers (OR 0.298, p < 0.001), in patients with a history of heavy alcohol use (OR 0.364, p < 0.001), and in patients with tumours of the floor of the mouth (OR 0.487, p = 0.013). Patients with anamnestic mucosal lesions had statistically significantly smaller tumours (Tis-T2 tumours OR 3.520, p < 0.001) and were referred to further care more often by oral health professionals (OR 2.081, p < 0.001) (Table 2). Previous diagnoses of dysplasia were statistically significantly less often detected in smokers (OR 0.329, p = 0.005) and in patients with a history of heavy alcohol use (OR 0.250, p = 0.024). In patients with previous dysplasia, OSCC was found incidentally statistically significantly more often than as a result of the patient actively seeking treatment (OR 6.009, p < 0.001). Dysplasia was statistically significantly associated with smaller tumours (OR 4.337, p = 0.007), and patients with preceding dysplasia were referred to further care more often by oral health professionals (OR 3.098, p = 0.022) (Table 3).

**Table 2:** Logistic regression model to explain the presence of previous oral mucosal findings with patient demographics, history of smoking and heavy alcohol use, tumour size, tumour site, referring physician, and care-seeking mode

Univariate	Multivariate
Previous oral mucosal finding present	Previous oral mucosal finding present
	95% CI for OR			95% CI for OR	
	OR	Lower	Upper	p-value		OR	Lower	Upper	p-value
**Age**	1.005	0.990	1.019	0.525	Heavy alcohol use				
Sex (reference male)					Yes	0.350	0.215	0.571	<0.001
Female	2.183	1.504	3.166	<0.001	Incidental findings				
**Smoking (reference non-smoker)**				Yes	2.671	1.704	4.187	<0.001
Current smoker	0.298	0.202	0.439	<0.001					
Heavy alcohol use									
Yes	0.364	0.225	0.587	<0.001					
**T-class (reference T3-T4)**									
Tis-T2	3.520	2.253	5.501	<0.001					
Site									
Tongue									
Yes	1.518	1.049	2.195	0.027					
**Gingiva**									
Yes	0.759	0.473	1.217	0.252					
**Floor of mouth**									
Yes	0.487	0.276	0.860	0.013					
**Palate**									
Yes	0.538	0.215	1.346	0.185					
Buccal									
Yes	2.039	1.065	3.904	0.032					
**Referring physician (reference other)**
General dentist, oral surgeon, or oral and maxillofacial surgeon	2.081	1.373	3.156	<0.001					
**Incidental findings**									
Yes	2.566	1.656	3.976	<0.001					


**Table 3:** Logistic regression model to explain the presence of previous oral mucosal dysplasia with patient demographics, history of smoking and heavy alcohol use, tumour size, tumour site, referring physician, and care-seeking type

Univariate	Multivariate
Previous oral mucosal dysplasia present	Previous oral mucosal dysplasia present
	95% CI for OR			95% CI for OR	
	OR	Lower	Upper	p-value		OR	Lower	Upper	p-value
**Age**	1.009	0.982	1.037	0.505	Heavy alcohol use				
Sex (reference male)					Yes	0.235	0.070	0.795	0.020
Female	1.122	0.557	2.261	0.747	Incidental findings				
**Smoking (reference non-smoker)**				Yes	6.195	3.004	12.774	< 0.001
Current smoker	0.329	0.151	0.720	0.005					
Heavy alcohol use									
Yes	0.250	0.075	0.830	0.024					
**T-class (reference T3-T4)**									
Tis-T2	4.337	1.504	12.508	0.007					
Site									
Tongue									
Yes	1.406	0.694	2.846	0.344					
**Gingiva**									
Yes	0.844	0.340	2.095	0.715					
**Floor of mouth**									
Yes	0.690	0.237	2.012	0.497					
**Palate**									
Yes	0.504	0.067	3.823	0.508					
**Buccal**									
Yes	1.195	0.349	4.096	0.776					
**Referring physician (reference other)**
General dentist, oral surgeon, or oral and maxillofacial surgeon	3.098	1.178	8.148	0.022					
**Incidental findings**									
Yes	6.009	2.937	12.294	< 0.001					


Both multiple logistic regression analyses for anamnestic oral mucosal lesions and previous oral mucosal dysplasia included the variables “heavy alcohol use” and “incidental finding”. OSCC in patients with a history of oral mucosal lesions was 2.7 times more often found incidentally (adjusted OR [aOR] 2.671, 95% confidence interval [CI] 1.704–4.187, p < 0.001). In addition, anamnesis of oral mucosal lesion was independently associated with heavy alcohol use (aOR 0.350, 95% CI 0.215–0.571, p < 0.001) (Table 2). OSCC in patients with previously detected dysplasia was less often found in patients with a history of heavy alcohol use (aOR 0.235, CI 0.070-0.795, p = 0.020) and was more often found incidentally (aOR 6.195, CI 3.004–12.774, p < 0.001) (Table 3).

## DISCUSSION

This study focused on earlier mucosal lesions of OSCC patients, patient profile, and care-seeking behaviour in relation to mucosal lesion history. We hypothesised that OSCC is often diagnosed in routine appointments in patients with a history of oral mucosal findings and previous oral mucosal dysplasia. Our hypothesis was confirmed. Suspicion of oral cancer was thus established more often in connection with a routine examination or an oral mucosal lesion follow-up visit. In addition, occurrence of earlier mucosal lesions was as high as 32.0% in OSCC patients. On the other hand, oral cancer is always preceded by a premalignant stage, but previous oral mucosal dysplasia was found in only 6.4% of these patients.^9,27,34^ These findings emphasise the need for more frequent identification of pre-malignant lesions.

Tumours of patients with a history of oral mucosal findings were more often Tis-T2 tumours and smaller than those of patients without preceding lesions. This is very likely the result of a follow-up of previous findings, which led to the early detection of OSCC. These patients did not necessarily have typical symptoms, such as pain, which would cause patients to seek treatment on their own, as described previously.^7,16,18,29,42^

In patients with a history of oral mucosal lesions, OSCC was more often located on the tongue and buccally. On the other hand, the floor of the mouth was less often the location of the earlier mucosal finding (Table 2). This is an interesting result, as the most typical locations for malignant findings in OSCC are the tongue and floor of the mouth.^3,24,32,35^ It is possible that clinicians more easily notice changes in the tongue and buccal mucosa and therefore these findings are more likely to be followed-up. Another explanation might be that cancer of the floor of the mouth is highly associated with alcohol use,^12^ which affects care-seeking, as we found. Treatment becomes increasingly challenging as the cancer progresses. Ensuring that these patients receive treatment before extensive tumour growth and spread is paramount. This could be accomplished by, for instance, public awareness campaigns, direct health advice given to this target group, and training of health professionals who meet these patients for oral examinations.

In our study, the most common observed previous lesion was the lichenoid-type reaction, which occurred in 12.5% of all OSCC patients (Fig 1) and in 20.0% of OSCC patients with previous oral mucosal dysplasia (Fig 2). The lichen planus is pathologically similar to the lichenoid reaction.^32,35^ Oral lichen planus and oral lichenoid reaction can both undergo malignant transformation. Malignant transformation may be linked to increased proliferative activity and decreased apoptosis rate of epithelial cells, which the inflammatory infiltrate may influence.^4^ Interestingly, in this study, clinically premalignant findings, oral leukoplakia and oral erythroplakia, were not the most commonly observed previous lesions in OSCC patients or in patients who had previous oral mucosal dysplasia.^20,26,28,33^ Because of these findings and the similarity of changes in the oral cavity, a biopsy is needed to confirm the diagnosis.^25,31^

In this study, dentists or oral and maxillofacial surgeons referred most of the patients (66.5%) for further OSCC care. These findings highlight the role of dentist, oral surgeon, or oral and maxillofacial surgeons in early OSCC diagnosis. The clinically important follow-up should be conducted by oral healthcare professionals in routine control appointments and in connection with other care.

The main limitation of this study is its retrospective design. Thus, information on the history of oral findings may have been missing due to incompleteness of the referral or because no preceding finding was made. However, based on our data, oral findings occurred in at least 32.0% of OSCC patients, and 6.4% of OSCC patients had previously-detected oral mucosal dysplasia. In addition, previous oral mucosal lesions were not always at the same site as the developing OSCC, and not all mucosal findings were premalignant. This is an important topic for further research, but larger and more treatment-oriented datasets are needed for comprehensive results.

## CONCLUSIONS

The findings of this study highlight the need for follow-up of oral mucosal changes for early detection of precancerous dysplasia, as only 6.4% of OSCC patients had prior diagnoses of oral mucosal dysplasia. This calls for thorough examinations and early biopsies, emphasising the crucial role of dentists in identifying premalignant lesions and OSCC. Notably, 66.5% of OSCC cases were referred by dental professionals, underscoring their importance in detecting asymptomatic changes to identify OSCC early.

## ACKNOWLEDGEMENTS

The Helsinki University Hospital Research Fund financially supported this work. Open access was funded by Helsinki University Library.

**Table 1:** Descriptive statistics for 528 patients with oral squamous cell carcinoma


**Age in years**		
Range	19–98	
Mean/Median	66.2/66.0	
	No. of patients	% of 528
**Sex**		
Male	309	58.5
Female	219	41.5
**Smoking**		
Non-smoker	261	49.4
Current smoker	267	50.6
**Heavy alcohol use**		
No	387	73.3
Yes	141	26.7
**T-class**		
Tis-T2	343	65.0
T3-4	185	35.0
**Site**		
Tongue	269	50.9
Gingiva	106	20.1
Floor of mouth	84	15.9
Palate	29	5.5
Buccal	40	7.6
**Referring physician**		
General dentist, oral surgeon, or oral and maxillofacial surgeon	351	66.5
Other	177	33.5
**Incidental findings**		
No	423	80.1
Yes	105	19.9
**Previous oral mucosal finding**		
No	359	68.0
Yes	169	32.0
**Previous oral mucosal dysplasia**		
No	494	93.6
Yes	34	6.4

